# Revisiting cefditoren for the treatment of community-acquired infections caused by human-adapted respiratory pathogens in adults

**DOI:** 10.1186/s40248-018-0152-5

**Published:** 2018-11-02

**Authors:** María-José Giménez, Lorenzo Aguilar, Juan José Granizo

**Affiliations:** 1 Research Department, PRISM-AG, Don Ramón de la Cruz 72, 28006 Madrid, Spain; 2 Preventive Medicine Department, Hospital Universitario Infanta Cristina, Parla, Madrid, Spain

## Abstract

Fifteen years after its licensure, this revision assesses the role of cefditoren facing the current pharmacoepidemiology of resistances in respiratory human-adapted pathogens (*Streptococcus pneumoniae*, *Streptococcus pyogenes*, *Haemophilus influenzae* and *Moraxella catarrhalis*). In the era of post- pneumococcal conjugate vaccines and in an environment of increasing diffusion of the *fts*I gene among *H. influenzae* isolates, published studies on the cefditoren in vitro microbiological activity, pharmacokinetic/pharmcodynamic (PK/PD) activity and clinical efficacy are reviewed. Based on published data, an overall analysis is performed for PK/PD susceptibility interpretation. Further translation of PK/PD data into clinical/microbiological outcomes obtained in clinical trials carried out in the respiratory indications approved for cefditoren in adults (tonsillitis, sinusitis, acute exacerbation of chronic bronchitis and community-acquired pneumonia) is commented. Finally, the role of cefditoren within the current antibiotic armamentarium for the treatment of community respiratory tract infections in adults is discussed based on the revised information on its intrinsic activity, pharmacodynamic adequacy and clinical/bacteriological efficacy. Cefditoren remains an option to be taken into account when selecting an oral antibiotic for the empirical treatment of respiratory infections in the community caused by human-adapted pathogens, even when considering changes in the pharmacoepidemiology of resistances over the last two decades.

## Key points

- Introduction of pneumococcal conjugate vaccines has modified the susceptibility profile of circulating *Streptococcus pneumoniae* in the community
- Antibiotic pressure in the community has facilitated the emergence and diffusion of β-lactamase negative ampicillin-resistant (BLNAR) and β-lactamase positive amoxicillin/clavulanate-resistant (BLPACR) *H. influenzae* isolates, implying resistance to several oral β-lactams
- The review of the high number of pharmacodynamic studies carried out with cefditoren since its licensure shows that cefditoren maintains its pharmacodynamic activity against the most prevalent bacterial isolates from community respiratory infections.

## Background

Among the complex niche representing the nasopharyngeal microbiota, four bacterial species have in common humans as exclusive commensals, with no animal or environmental reservoirs contributing to their life-cycle: *Streptococcus pneumoniae*, *Streptococcus pyogenes*, *Haemophilus influenzae* and *Moraxella catarrhalis*, with different turnover rates in nasopharynx. Human to human transmission, which occurs via respiratory droplets, is critical for them to persist. Alterations in the ecological niche or migrations to other niches are responsible for their change to human-adapted pathogens causing pharyngotonsillitis (*S. pyogenes*) or otitis, rhinosinusitis and lower respiratory tract infections (LRTIs) (*S. pneumoniae*, *H. influenzae* and *M. catarrhalis*). Thus, apart from their commensalism, they exhibit direct pathogenicity.

Additionally, the indirect pathogenicity of *H. influenzae* and *M. catarrhalis* protecting *S. pyogenes* and *S. pneumoniae* from the action of some β-lactam antibiotics by means of their β-lactamases has been described. In vivo, formation of biofilms (which are larger when *S. pneumoniae* and *H. influenzae* or *M. catarrhalis* are present than when only one species is alone) [1] favors indirect pathogenicity and intracellular antibiotic deactivation [2]. The coexistence of susceptible and resistant cells within these bacterial communities increases the opportunity for horizontal gene transfer during antibiotic selection pressure [2]. This gene transfer depends on the duration of carriage: strains with longer duration of carriage have a greater risk of antibiotic exposure and thus, greater risk for acquiring resistance [3]. In turn, resistance implies fitness advantages for bacteria in the presence of antibiotics, favoring spread of resistant isolates within the community.

Antibiotic resistance significantly impacts on patient’s illness burden in the community, and patients with laboratory-confirmed antibiotic-resistant respiratory tract infections (RTIs) are likely to experience delayed recovery following antibiotic treatment [4]. In the era prior to licensure of conjugate pneumococcal vaccines (PCV), antibiotic use was the basic and exclusive force behind resistance patterns in bacteria isolated from community-acquired infections [5–7] despite of descriptions of correlations between pneumococcal resistance and educational level, climate and proportion of young people in the population [5]. At different geographical areas, positive correlations between percentages of macrolide resistance in *S. pneumoniae* and *S. pyogenes* were described [8], and resistance was associated with macrolide consumption (mainly compounds exhibiting long half-life) [7, 9, 10]. Similarly, β-lactam consumption (mainly oral 2^nd^ generation cephalosporins) was associated with penicillin resistance in *S. pneumoniae* [9]. A global ecological relationship of resistance between penicillin-resistant *S. pneumoniae*, erythromycin-resistant *S. pneumoniae*, erythromycin-resistant *S. pyogenes* and ampicillin-resistant *H. influenzae* was described [11], reinforcing the idea of consumption of certain antibiotics as driver of resistances in human-adapted respiratory pathogens in the community.

In the post-vaccine era, this situation has completely changed with a decrease in the prevalence of penicillin-resistant *S. pneumoniae* (with lower changes in erythromycin resistance in both streptococcal species) and the emergence of ampicillin-resistant phenotypes not related to β-lactamase production in *H. influenzae*. These complex dynamics have been influenced by different factors in the present century: vaccine pressure and natural serotypes fluctuations, co-selection of resistances, selection of co-resistances, and antibiotic consumption patterns according to the population structure [12], among others.

**Fig. 1:**
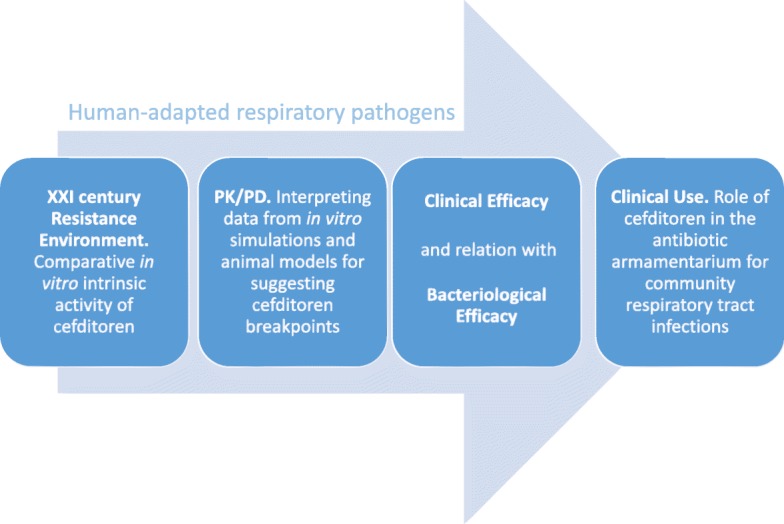
Cefditoren: steps analyzed from Microbiology to Clinical Use

Figure 1 shows the steps analyzed in this extensive revision.

## Pharmacoepidemiology of resistances in human-adapted respiratory pathogens

Although they have in common being human-adapted pathogens colonizing the nasopharynx as their natural ecological niche, not all of them have equally responded (on the basis of non-susceptibility emergence and dissemination) to the preventive and therapeutic measures introduced. Spread of resistance in *S. pyogenes* and *M. catarrhalis*, limited to certain antibiotics, contrasts with emergence and spread of resistance to β-lactams and macrolides in *S. pneumoniae* and *H. influenzae*, driving to more complex scenarios.

*S. pyogenes* is uniformly susceptible to β-lactams and, among oral cephalosporins, cefditoren exhibits the highest intrinsic activity, with MIC_90_ values ≤0.06 mg/l. [13–15] Resistance to erythromycin (implying resistance to clarithromycin and azithromycin) varies from < 10% (USA, Baltic countries, Romania), to 10–20% (Poland, Czech Republic, Spain) and 25–35% (Slovakia, Hungary, Hong Kong), being up to 93.5% in China [13, 16–20].

Almost 100% strains of *M. catarrhalis* are β-lactamase producers [21]; MIC_90_ values of cefditoren (range from 0.25 to 0.5 mg/l) are lower than those of amoxicillin/clavulanic acid (range from 0.25 to 2 mg/l) and cefuroxime (range from 2 to 8 mg/l [14, 15, 22, 23].

### *Streptococcus pneumoniae*: the post-PCV13 scenario

Based on the most common pediatric serotypes, PCV7 was developed and introduced for universal immunization of healthy children in most developed countries in 2000–1. However, detection of emerging new serotypes in the late PCV7 period led to the inclusion of six additional serotypes in the new PCV13 in 2010. Since then, decreases in invasive pneumococcal disease (IPD), not only in children but also in adults due to herd effect [24–27], as well as in PCV13 serotypes involved in non-invasive diseases, have been described [25, 27]. Although some studies [28–30], but not others [24, 31], have found slight increases in non-PCV13 serotypes causing IPD, individually none of the non-PCV13 serotypes has shown a significant increase [32], in contrast to the increase in serotype 19A (with its associated resistant sequence types [33]) after PCV7 implementation [33–35].

Since susceptibility is strongly linked to serotypes, targeting prevalent capsular serotypes with PCVs is extremely effective in reducing resistant infections [36]. While in countries that lack a significant PCV coverage > 40% isolates are penicillin-resistant [36], in those where PCV13 was introduced, antibiotic–nonsusceptible IPD has decreased in all age groups [24, 25, 37], as well as antibiotic–nonsusceptible isolates colonizing the nasopharynx in children [38]. Data from 2013 in USA showed that the proportions of non-susceptible IPD cases were around 10% for penicillin and 33% for macrolides in children, and around 6 and 28%, respectively, in adults [37]. These high nonsusceptibility rates to macrolides in USA are lower than those found in a surveillance from 11 Asian countries reporting a mean percentage of resistance to erythromycin as high as 72.7%, with the highest rates in China (96.4%), Taiwan (84.9%) and Vietnam (80.7%) [39]. Another report from Thailand confirmed high resistance rates to erythromycin (51.3%) [40] in Asia.

Few pneumococcal surveillance studies including cefditoren among the antimicrobials tested have been published in the last 5 years. Studies published in the previous decade (2007–2010) (pre-PCV13 era) showed that among penicillin-susceptible isolates, cefditoren MIC_90_ values ranged from ≤0.03 to 0.06 mg/l. [14, 15, 41–48] Against penicillin-intermediate strains, values increased to 0.25–0.5 mg/l while among penicillin-resistant strains, they ranged from 0.5 to 1 mg/l. [14, 15, 41–48] For amoxicillin and cefuroxime, MIC_90_ values for penicillin-susceptible isolates ranged from 0.12 to 0.25 mg/l [14, 15, 41–47] for cefuroxime and from 0.03 to 0.25 mg/l for amoxicillin [14, 15, 42–47]. For penicillin-intermediate strains, values ranged from 4 to 8 mg/l [14, 15, 41–47] for cefuroxime and from 1 to 4 mg/l for amoxicillin [14, 15, 42–47], while for penicillin-resistant strains, they ranged from 8 to 32 mg/l for cefuroxime and from 8 to ≥16 mg/l for amoxicillin [14, 15, 41–47]. All these studies showed that cefditoren exhibited the highest in vitro intrinsic activity compared with other oral β-lactams [14, 15, 41–48]. Other studies in the last decade not distributing strains by penicillin susceptibility also showed MIC_50_/MIC_90_ values of cefditoren lower than those of other oral cephalosporins: ≤0.06 and 1 mg/l, respectively, in a study in 11 Asian countries [49], and ≤ 0.015 and 0.125 mg/l, respectively, in a Spanish surveillance [16]. The last surveillance study (including cefditoren among the antimicrobials tested) found in the literature, a surveillance study in Japan, showed MIC_50_/MIC_90_ values of cefditoren of 0.125/0.5 mg/l. [50]

The highest activity of cefditoren was also demonstrated in an in vitro study comparing cidal activity of serum achievable concentrations of third-generation oral cephalosporins (cefditoren, cefpodoxime, cefdinir, and cefixime), using cefotaxime as control drug, against pneumococcal strains distributed by cefotaxime MICs (0.5 to 2 mg/l) [51]. Against strains with cefotaxime MIC > 1 mg/l, only cefditoren (at 0.5–1 mg/l concentrations, which are lower than the maximum concentration in serum) achieved > 90% reduction of initial inocula [51]. Another study comparing cidal activity of cefditoren with that of other β-lactams (amoxicillin/clavulanic acid, cefuroxime, cefixime and cefpodoxime) and levofloxacin showed that cefditoren was the only agent achieving, at concentrations equal to 2× MIC, significant bactericidal activity (≥3 log reduction of initial inocula within 4 h) against penicillin- susceptible and resistant *S. pneumoniae* [52].

These studies, carried out in the pre-PCV13 era, demonstrated the high comparative intrinsic activity (in terms of MIC and bactericidal activity) of cefditoren against *S. pneumoniae*. In the post-PCV13 era, considering that, as stated, the vaccine implementation has reduced the prevalence of pneumococcal resistance in the community [24, 25, 36–38] by targeting most resistant serotypes, the number of penicillin-resistant isolates with high cefditoren MICs (i.e > 0.25 mg/l) should had necessarily been reduced.

### *Haemophilus influenzae*

Traditionally, β-lactamase production was the mechanism of resistance to aminopenicillins in non-typeable *H. influenzae* (NTHi). However, antibiotic pressure by amoxicillin/clavulanate and oral cephalosporins contributed to the spread of non-enzymatic resistance mechanisms linked to the *fts*I gene [53]. In the past decade, many countries experimented a rapid increase in genetically-defined β-lactamase negative ampicillin-resistant (gBLNAR) isolates, accounting for 15–30% of all isolates, among others, in Europe, Australia and USA, with up to 50% in Japan [54–59]. In Spain BLNAR isolates increased in parallel with decreases in β-lactamase producing-isolates [60], and the last published study determining susceptibility of invasive *H. influenzae* isolates showed up to 19% gBLNAR isolates compared with 16.9% isolates resistant due to β-lactamase production [61]. Similarly, a recent report from Japan showed the lower percentage of ampicillin-resistant strains due to β-lactamase production (5.6%) than to BLNAR (37.2%) among NTHi respiratory isolates [50]. In Sweden, the increase in β-lactam resistance among invasive *H. influenzae* isolates has also been attributed to the significant increase in BLNAR isolates [62].

Isolates exhibiting both mechanisms of resistance (β-lactamase production and mutations in the *fts*I gene), the so-called β-lactamase positive amoxicillin/clavulanic acid-resistant (BLPACR) isolates, account for lower proportions than BLNAR isolates [16, 58, 60], also among invasive isolates [61], generally with percentages lower than 5%. However, higher and overtime constant rates of BLPACR isolates [59] and marked increases from 2011 to 2013, reaching 19% in China [63], have been reported.

Published data show that both against β-lactamase positive and negative NTHi isolates, MIC_90_ values of cefditoren (range from ≤0.03 to 0.06 mg/l) are 5–7 dilutions lower than those of amoxicillin/clavulanic acid or cefuroxime (MIC_90_ range from 1 to 8 mg/l for both compounds) [14, 15, 22, 41, 44]. PBP3 modifications subsequent to *fts*I gene mutations could decrease susceptibility not only to aminopenicillins but also to some oral cephalosporins as cefaclor or cefuroxime [60, 61, 64]. Against BLNAR isolates MIC_90_ values were 0.03–0.06 mg/l for cefditoren; values markedly lower than those for amoxicillin/clavulanic acid (4 mg/l) and for cefuroxime (2–16 mg/l) [14, 15, 65]. Recent reports have confirmed the high intrinsic activity of cefditoren against NTHi independently of the production of β-lactamase or the BLNAR phenotype [66, 67] or the presence of both resistance mechanisms [65]. On the other hand, NTHi can be considered intrinsically resistant to macrolides, being its resistance associated with the presence of efflux pumps in virtually all strains [68].

Table 1 resumes the comparative intrinsic activity of cefditoren in terms of MIC_50_/MIC_90_ values obtained in the different studies.

**Table 1:** Comparative intrinsic activity: Range (mg/l) of MIC_50_ and MIC_90_ from published studies

	References	Amoxicillin/clavulanic acid		Cefuroxime		Cefditoren	
		MIC_50_	MIC_90_	MIC_50_	MIC_90_	MIC_50_	MIC_90_
*Streptococcus pyogenes* ^a^	13–15	≤0.012–0.06	≤0.012–0.12	0.03–0.06	0.12	≤0.03	≤0.03–0.06
*Streptococcus pneumoniae* ^b^	14,15,41–48						
Penicillin-susceptible		≤0.015–0.06	0.03–0.25	≤0.03	0.12–0.25	≤0.015	≤0.03–0.06
Penicillin-intermediate		0.25–1	1–4	0.5–4	4–8	0.06–0.25	0.25–0.5
Penicillin-resistant		2 - ≥16	8 - ≥16	4–8	8–32	0.25–0.5	0.5–1
*Haemophilus influenzae*							
β-lactamase negative	14,15,22,41,44	0.25–1	1–8	0.25–2	1–8	≤0.08	≤0.03–0.06
β-lactamase positive	14,15,22,41,44	0.5–2	2–4	1–2	2–8	≤0.08	≤0.03–0.06
BLNAR	14,15,65	2	4	0.5–4	2–16	≤0.08	0.03–0.06
BLPACR	65	4	8	4	16	0.03	0.06
*Moraxella catarrhalis* (β-lactamase positive)	14,15,22,23	0.12–0.5	0.25–2	1	2–8	0.06–0.12	0.25–0.5

^a^In terms of ampicillin (ref. [13])

^b^In terms of amoxicillin (ref. [14, 15, 43–46])

## Revisiting cefditoren pharmacokinetic/pharmacodynamic (PK/PD) data to interpret susceptibility [69–71]

Fortunately, cefditoren is a drug with high number of pharmacodynamic studies published in the literature. Pharmacodynamics constitute one of the major basis for setting breakpoints by Regulatory Agencies [72]. For this reason, and since there are not established CLSI or EUCAST breakpoints for cefditoren to guide interpretation of susceptibility data, for clinical decision making, the analysis of published pharmacodynamic data is essential to delimit susceptibility. Table 2 shows a summary of the PK/PD studies performed with cefditoren [73–85]. Most of the studies carried out against *S. pneumoniae* included isolates exhibiting a wide range of cefditoren MIC values (from 0.125 to 4 mg/l). For PK/PD interpretation, these pneumococcal studies acquire relevance because in the studies carried out with *H. influenzae* or *S. pyogenes* it was not possible to test the wide range of MIC values required for evaluation, due to the very high intrinsic activity of cefditoren against both species, as has been commented. Importantly, some of the pharmacodynamic studies addressed the high protein binding rate of cefditoren, which is ≈88% [86]. Through the addition of human serum or albumin in the tubes/devices used in in vitro tests [73–75] or animal models in mice (where the experimentally measured protein binding rate, 87%, was similar to the rate in humans) [84], pharmacodynamics were determined in more realistic situations than using extrapolated free drug concentrations.

**Table 2:** Summary of PK/PD studies carried out with cefditoren (CDN)

Ref.	Type of study	Strains	Comparators	Main conclusion
[73]	Killing curves in the presence/absence of human albumin (Cmax: 4.1 mg/l)	*S. pneumoniae* (CDN MICs: 0.12–0.5 mg/l)	_	The activity of cefditoren should not be linked exclusively with the theoretical unbound fraction extrapolated from the plasma concentration.
[74]	Killing curves in the presence/absence of human albumin or human serum (Cmax: 4.1 mg/l)	*S. pneumoniae* (CDN MICs: 0.12–0.5 mg/l)	_	The presence of 90% human serum did not limit bactericidal activity as did the use of concentrations similar to free-drug.
[75]	In vitro computerized pharmacodynamic simulation in the presence of 75% human serum	*S. pneumoniae* (CDN MICs: 0.25–0.5 mg/l)	_	Cefditoren physiological concentrations exerted antibacterial activity against strains exhibiting MICs of 0.25 and 0.5 m/l under protein binding conditions similar to those in humans (experimentally measured)
[76]	In vitro computerized pharmacodynamic simulation (total vs. free concentrations)	*H. influenzae* (including BLNAR and BLPACR)	Co-amoxiclav	The experimental bactericidal activity of cefditoren (both total and free concentrations) was maintained over the dosing interval regardless of the presence of mutation in the *fts*I gene or β-lactamase production, in contrast to co-amoxiclav.
[77]	In vitro computerized pharmacodynamic simulation	*S. pneumoniae* (amoxicillin MIC > penicillin MIC) (CDN MICs: 0.12–1 mg/l)	Cefuroxime Co-amoxiclav	Bactericidal activity at 12 and 24 h was obtained against all strains with cefditoren, but not with comparators.
[78]	In vitro computerized pharmacodynamic simulation	*S. pneumoniae* (mixed inocula) (CDN MICs: 0.015, 0.5, 1 mg/l)	Cefuroxime Cefixime Cefaclor Amoxicillin	Against penicillin resistant strains, cefditoren (but not comparators) decreased the initial bacterial load all along the simulation, without regrowth and with lower selection of resistant subpopulations
[79]	In vitro computerized pharmacodynamic simulation	*H. influenzae* (including BLNAR and BLPACR)	Cefuroxime Co-amoxiclav	Cefditoren exhibited the highest bactericidal activity maintained over time against ampicillin-resistant *H. influenzae*, regardless of beta-lactamase production and/or BLNAR phenotype.
[80]	In vitro computerized pharmacodynamic simulation	*H. influenzae* β^−^ *H. influenzae* β+ BLNAR BLPACR (mixed inocula)	Cefuroxime Co-amoxiclav	Cefditoren offered higher antibacterial effect than comparators due to its higher activity against beta-lactamase-producing strains and those carrying *fts*I gene mutations. BLNAR and BLPACR strains were selected by cefuroxime and co-amoxiclav, respectively.
[81]	In vitro computerized pharmacodynamic simulation	*S. pyogenes* *S. pneumoniae* *H. influenzae* β+ BLPACR (mixed inocula)	Amoxicillin Co-amoxclav	Cefditoren (but not comparators) completely countered indirect pathogenicity and eradicated *S. pyogenes* and both *H. influenzae* strains.
[82]	In vitro computerized pharmacodynamic simulation in media containing *fts*I DNA	*H. influenzae* β^−^ *H. influenzae* β+	Co-amoxiclav	Cefditoren (but not co-amoxiclav) was bactericidal and countered intrastrain *fts*I gene diffusion
[83]	In vitro study assessing by flow cytometry the deposition/binding of components of the complement system to bacterial cells	*S. pneumoniae*	Ceftriaxone	Increased recognition of *S. pneumoniae* by the complement system in the presence of sub-inhibitory concentrations of cefditoren
[84]	Mice sepsis model (pre-immunized vs. non pre-immunized mice)	*S. pneumoniae* (CDN MICs: 1, 2, 4 mg/l)	_	In non pre-immunized animals, t > MIC values for CDN of approximately 35% (total) and approximately 19% (free) were associated with 100% survival, with lower values in pre-immunized animals
[85]	Monte Carlo simulation	_	_	Coverage with total concentrations: a. criterion of 40% t > MIC: MICs ≤0.5 mg/l b. criterion of 33% t > MIC: MICs ≤0.5 mg/l Coverage with extrapolated free concentrations: a. criterion of 40% t > MIC: MICs ≤0.12 mg/l b. criterion of 33% t > MIC: MICs ≤0.25 mg/l

One remarkable fact of cefditoren pharmacokinetics is the different bioavailability in the fast/fed states. The administration of cefditoren-pivoxil following a high fat meal results in a 70% increase in mean AUC and 50% increase in mean Cmax compared to administration of cefditoren-pivoxil in the fasted state [87]. Therefore, conclusions of pharmacodynamic studies are different depending on the fed/fast condition of subjects included [85, 88], and for this reason the prescribing information recommends cefditoren intake with meals to enhance absorption [87].

Essential points guiding susceptibility interpretation are the accepted cut-off values of the PK/PD index predicting efficacy for β-lactams (percentage of dosing interval that antibiotic concentrations exceed the MIC; t > MIC), set at 40% for clinical cure and at 33% for bacteriostasis [89, 90]. The FDA and CLSI definitions of the “susceptible” category involve the bacteriostatic endpoint (33% t > MIC) since “susceptibility” is defined as the likely *inhibition* of the pathogen if the antimicrobial compound reaches the concentration usually achievable after administration of the recommended dose [91, 92].

Several susceptibility breakpoints have been proposed for cefditoren [93–95], ranging from ≤0.125 mg/l (included in the FDA prescribing information) [87] to ≤0.5 mg/l (approved by the Reference Member State, Spain, during the Mutual Recognition Procedure in Europe) [96]. In vivo, a pneumococcal sepsis model infecting mice with isolates exhibiting exceptionally high MICs of cefditoren (1–2 mg/l) exceeding the proposed susceptibility breakpoints, showed that cefditoren t > MIC of ≈35% (free t > MIC of ≈20%) produced 100% survival compared with 0% survival in untreated animals [84]. A free t > MIC value of ≈20% was also related to > 99.9% reduction in bacterial load of two different pneumococcal isolates (MICs of 0.25 μg/ml) in an in vitro pharmacodynamic simulation with physiological albumin concentrations and 86% protein binding rate in the device [75]. These two studies demonstrated that the limitation of the activity of cefditoren by protein binding is far from absolute, being the activity of total concentrations in the presence of albumin/serum higher than the one exhibited by calculated free concentrations.

The range of proposed breakpoints (from ≤0.125 to ≤0.5 mg/l) is well supported by a Monte Carlo simulation of cefditoren investigating coverage by concentrations achieved in serum after 400 mg oral dosing (after meals) [97] according to different criteria: a) By using total concentrations and the bacteriostatic criterion (33% t > MIC), cefditoren covered strains inhibited by MICs up to 0.5 mg/l, b) By using extrapolated free drug concentrations and 33% t > MIC, coverage was achieved for strains inhibited by MICs up to 0.25 mg/l, and c) By using extrapolated free drug concentrations and the criterion of 40% t > MIC, strains exhibiting MICs up to 0.12 mg/l were covered [85].

Evidently, the application of one or another criteria does not affect susceptibility rates of *H. influenzae* and *S. pyogenes* since even by applying the strictest PK/PD breakpoint for cefditoren (≤0.125 mg/l), susceptibility rates were almost 100% [16, 22, 41]. With respect to *S. pneumoniae*, Table 3 shows ranges of percentages of susceptibility against penicillin-susceptible (MIC of penicillin ≤0.06 mg/l), penicillin-intermediate and penicillin-resistant (MIC of penicillin ≥2 mg/l) pneumococcal isolates, calculated with MIC distributions from published studies [41–45] by applying CLSI breakpoints for amoxicillin and cefuroxime, and the three proposed breakpoints for cefditoren. As observed, the decrease in susceptibility to penicillin affects more the susceptibility to cefuroxime than the susceptibility to amoxicillin. For the susceptibility to cefditoren, the impact of penicillin non-susceptibility is dependent on the breakpoint value considered: high for ≤0.125 mg/l but negligible for ≤0.5 mg/l (with 95% isolates susceptible to cefditoren whether they were penicillin- susceptible, intermediate or resistant). This illustrates the importance of having breakpoints for susceptibility interpretation of microbiological data.

**Table 3:** Ranges of susceptibility against penicillin-susceptible (≤0.06 mg/l), penicillin-intermediate and penicillin-resistant (≥2 mg/l) pneumococcal isolates, calculated with MIC distributions from published studies [41–45] by applying CLSI breakpoints (amoxicillin, cefuroxime) and the three proposed breakpoints for cefditoren

Antibiotic	Penicillin susceptible isolates	Penicillin intermediate isolates	Penicillin resistant isolates
Amoxicillin^a^ (≤2 mg/l)	100%	78.4–100%	17.5–76%
Cefuroxime (≤1 mg/l)	99.7–100%	33.8–67.6%	0–0.4%
Cefditoren			
≤ 0.125 mg/l	99.1–100%	37.2–71.2%	0–0.4%
≤ 0.25 mg/l	100%	67.6–88.6%	15.2–57.3%
≤ 0.5 mg/l	100%	99.3–100%	95.3–100%

^a^Amoxicillin/clavulanic acid in references [41, 42]

## From PK/PD interpretation to clinical data in adults

Pharmacodynamic breakpoints can be related with bacterial eradication and subsequent therapeutic outcome [98]. For this reason, microbiological evaluation in clinical trials provides the best quality data to support breakpoints. However, despite the percentage of resistance in the community, in a clinical trial it is difficult to have enough number of patients infected by resistant bacteria to reach conclusions. To overcome this, data from microbiological evaluation in clinical trials of community-acquired pneumonia (CAP) and acute exacerbations of chronic bronchitis (AECB) carried out with cefditoren were pooled and analyzed in order to increase the number of evaluable patients. In that analysis, 100% penicillin non-susceptible (MIC ≥0.12 mg/l) isolates of *S. pneumoniae* in the cefditoren 400 mg group (*n* = 20), 16 of 19 (84.2%) strains in the 200 mg group, and 16 of 17 (94.1%) strains in the comparator group were eradicated or presumably eradicated [99]. Among *S. pneumoniae* isolates showing penicillin resistance (MIC ≥2 mg/l), 17 out of 18 (94.4%) isolates from patients in both cefditoren arms were eradicated or presumably eradicated compared with 10 out of 11 (90.9%) in the comparator group [99].

This microbiological efficacy was accompanied by clinical efficacy, with cefditoren showing similar percentages of clinical cure than antimicrobials used as comparators. Table 4 summarizes published clinical outcomes with cefditoren in clinical trials of the different approved respiratory indications [99–109].

**Table 4:** Percentage of responders in the clinical evaluation of cefditoren efficacy in clinical trials. Published data

	References	Cefditoren 200 mg bid		Cefditoren 400 mg bid		Comparators^a^	
		EOT^b^	EF^c^	EOT^b^	EF^c^	EOT^b^	EF^c^
Pharyngotonsillitis	90-92	93.4 – 98.6	88.9–99.2	–	–	88.6–97.1	84.4–100
Rhinosinusitis	90,93	81.3–95.2	63.6–91.0	76.5–83.1	70.2–71.9	75.5–97.7	66.2–95.1
AECB^d^	89,94–97	80–88.8	79.7–83.0	84.4–95.5	78.1–85.6	75.0–98.9	79.8–85.7
CAP^e^	89,98,99	87.2 – 91.8	88.4–87.8	89.2–90.1	83.7–87.2	90.3–92.2	87.8–93.8

^a^Penicillin V or VK (90–92) for pharyngotonsillitis, amoxicillin/clavulanic acid (90,93) or cefuroxime (90) for rhinosinusitis, cefuroxime (89,94,96) or clarithromycin (89,95) for AECB, and amoxicillin/clavulanic acid (89,98), and cefpodoxime (89,99) for CAP

^b^EOT = End of Treatment

^c^EF = End of Follow up

^d^AECB = Acute exacerbation of chronic bronchitis

^e^CAP = Community-acquired pneumonia

In pharyngotonsillitis studies, since all patients included in clinical trials had a pre-treatment positive culture, the relationship between the microbiological and clinical response obtained was investigated. The percentage of favorable clinical response was significantly higher (*p* < 0.01) in patients showing bacteriological eradication (≥98.5%) than in those showing bacteriological persistence (≤51.4%) [100], evidencing the relationship between microbiological and clinical responses. Overall, the post-treatment microbiological response (eradication) was significantly (*p* < 0.01) higher with cefditoren versus penicillin (used as comparator): 90.4% versus 82.7% at the end-of-treatment visit, and 84.7% versus 76.7% at the end of follow up visit, respectively [100]. In a recent revision of eight published clinical studies, failure rates with penicillin in the treatment of pharyngotonsillitis ranged from 14 to 40%, and one of the main reasons advocated by the author was penicillin inactivation by β-lactamases present in the environment produced by β-lactamase producing bacteria [110], as *H. influenzae*. This “indirect pathogenicity” was demonstrated in a pharmacodynamic simulation using a mixed inocula of *S. pyogenes*, penicillin-resistant *S. pneumoniae*, β-lactamase positive NTHi and a BLPACR strain exposed to simulated serum concentrations of amoxicillin, amoxicillin/clavulanic acid or cefditoren [81]. Of the three compounds, cefditoren, stable to β-lactamases was the unique compound that completely countered the indirect pathogenicity and eradicated both β-lactamase positive organisms [81]. For the BLPACR strain the accumulation of two resistance traits (β-lactamase production and *fts*I gene) represented a competitive advantage in the presence of amoxicillin/clavulanic acid [82], this compound not being able to completely counter indirect pathogenicity as cefditoren did. These data acquire relevance since antibiotic-treated individuals are sources for spread of β-lactamase producing bacteria to other individuals [110], and carriage of *H. influenzae* has been associated with vaccination with PCVs [111].

Antibiotic degradation via β-lactamases enabling growth of susceptible cells in their vicinity [2, 112], even across species, has been demonstrated in other in vitro studies and in animal models, favoring *S. pneumoniae* growth in the presence of *H. influenzae* or *M. catarrhalis* [1, 2, 113]. In the context of biofilms, interactions between species are facilitated, and this may explain the tendency of different species to occur together [114], as *H. influenzae* and *S. pneumoniae*, which form a much larger biofilm together than either bacterium does on its own [115]. This is one of the reasons for the increasing interest on biofilms in the investigational field. One of the major goals in modern clinical microbiology is the development of strategies capable of reducing biofilm infections related to chronic conditions due to the difficulty for antibiotics in eradicating bacteria within these structures (due to difficulties in reaching bacterial cells or diminished bacterial growth rate, leading to persistence). Microorganisms typically colonizing the human respiratory tract make full use of the biofilm strategy when causing non-invasive disease (chronic bronchitis, sinusitis and otitis).

In chronic bronchitis, antibiotic therapy overcomes the symptoms caused by waves of planktonic cells released from the biofilm during exacerbations of the disease but fails to eradicate infection as sessile cells are inherently less affected [116]. Due to these facts not only time to eradication of symptoms is an endpoint in clinical trials with antibiotics against exacerbations, but also time to relapse is increasingly used as endpoint. In healthy lungs, there is a transient microbiome of micro-aspirated upper airway microbial flora being cleared by normal lung defense mechanisms [117]. Chronic exposure to cigarette smoke results in higher loads of NTHi and *S. pneumoniae* in lungs [118], and these potential pathogenic microorganisms have been detected in approximately 25% of patients with chronic obstructive pulmonary disease (COPD) during stable disease [119]. COPD and subsequent AECBs (with related presence of released planktonic cells) are associated with biofilms [120–123], with a relevant role for *H. influenzae*. In addition, COPD patients with frequent exacerbations show an increase in inflammation in the upper airways contributing to the progression of the disease [124]. Antibiotics active against planktonic cells and able to interfere or decrease biofilm development may offer clinical advantages [125]. Additionally, the use of antibiotics may also reduce inflammatory parameters [107]. Cefditoren showed to interfere biofilm formation in a study comparing cefditoren (0.03 mg/l) with amoxicillin/clavulanic acid (1/0.5 mg/l) that concluded that both compounds were able to reduce biofilm formation by the 10 pneumococcal isolates tested, with significant higher reductions in the case of cefditoren [126]. With respect to inflammation, one study comparing cefditoren and levofloxacin found that the use of both antimicrobials was associated with significant reductions of IL-6 and KL-6, two mediators of lung inflammation and epithelial damage [107, 127].

The acquisition of a new strain of *H. influenzae* (responsible for 13–50% of AECBs [128, 129]), *S. pneumoniae* or *M. catarrhalis* has been described as a fact increasing the risk of exacerbation [130]. With respect to *S. pneumoniae*, it has been reported that new episodes occurring within the first 3 months after a previous episode show a high probability of being caused by the same strain, an important feature for election of empiric therapy [131]. Based on its in vitro activity against human-associated respiratory pathogens (Table 1) and concentrations in bronchial mucosa of 0.56–1.04 mg/kg [127], cefditoren provides adequate focal coverage in AECB, as demonstrated by clinical trials (Table 4). One of the clinical trials of AECB treated with cefditoren investigated the relationship between the clinical and bacteriological response, and suggested that the response to the antibiotic was more rapidly seen in signs that depend on the bacterial location (more rapid and greater decrease over time in sputum volume, and purulence and rales and rhonchi) than in those depending in part on previous structural damage [104]. In this trial, clinical success by key baseline pathogen was 84.0% vs. 82.5% for NTHi, and 92.3% vs. 81.3% for *S. pneumoniae* for cefditoren vs. cefuroxime, respectively [104].

Another mixed infection (mainly, *S. pneumoniae* and *H. influenzae*) involving biofilms is rhinosinusitis, where cefditoren has shown percentages of clinical efficacy similar to those of AECB at the end of treatment in clinical trials (Table 4).

## The role of cefditoren in the treatment of LRTIs

Further non-clinical studies have corroborated the adequacy of cefditoren in the treatment of LRTIs. The TOM probability model was used to predict the likelihood of clinical success in AECB considering, among other factors, the likelihood of spontaneous resolution [132]. The study concluded that fluoroquinolones, cefditoren and high doses of amoxicillin/clavulanic acid were the antimicrobials that highest predict clinical efficacy in the treatment of AECB in contrast to cefaclor and macrolides (with predictions not much higher than that of placebo) [132]. These results are in accordance with a 15-year longitudinal study of COPD showing that macrolides were ineffective in eradicating *H. influenzae* [133].

Using the Delphi methodology to assess the consensus on the most appropriate strategy for acute LRTIs, 71% agreement was reached on the statement that empiric therapy with antibiotics characterized by a resistance profile above the 10–20% threshold and the non-protected β-lactams should be avoided [134]. This, together with the TOM probability study [132] points to discard macrolides as treatment considering their lack of PK/PD activity against *H. influenzae*, a pathogen potentially involved. Consensus (78% agreement) was also reached on the need to use antibiotics able to target emergent BLNAR and BLPACR isolates to prevent intra-species diffusion of resistant strains when treating AECB [134]. In vitro, cefditoren (in contrast to amoxicillin/clavulanic acid) demonstrated to counter intra-strain diffusion and spread of nonenzymatic resistance mechanisms (*fts*I gene) in a pharmacodynamic simulation including several NTHi strains [82]. Consensus (96% agreement) was also reached on the statement that among third-generation cephalosporins, cefditoren has a particularly balanced spectrum, the 400 mg dose every 12 h covering penicillin-resistant pneumococci [134].

Apart from the use of cefditoren in the treatment of mild to moderate CAP [87, 96], cefditoren may have a role as oral treatment following intravenous treatment with third generation cephalosporins. Among others, IDSA and ATS guidelines recommend a switch to oral antibiotics for the treatment of stable hospitalized patients with CAP as soon as the patient is improving [135, 136]. A very high consensus (93%) was reached in the Delphi-based analysis on the statement that cefditoren is the best switch for intravenous third generation cephalosporins (cefotaxime, ceftriaxone) because of the similar spectrum and the highest intrinsic activity [134]. In this sense, cefditoren has been included as recommended drug for switch therapy after intravenous treatment with third generation cephalosporins in several documents [137–139], in contrast to cefuroxime which does not provide the required high intrinsic activity and adequate pharmacodynamics [139].

In a meta-analysis of six randomized clinical trials including 1219 patients hospitalized with moderate to severe CAP, the authors concluded that early conversion to oral antibacterial treatment appeared to be as effective as traditional intravenous treatment and was associated with fewer drug-related adverse events and shorter hospital stays [140], thus also reducing associated costs. In the treatment of LRTIs, the price of the antibiotic therapy does not represent the most affecting health care cost. In the Delphi-based approach, high consensus was reached on the statement that the price of the initial antibiotic therapy does not represent the most affecting health care cost (88% consensus) [134]. Switch from parenteral to oral antibiotic therapy reduces length and costs of hospitalization, and the risk of hospital-acquired infections, improving patient’s quality of life as well (100% agreement) [134]. On the contrary, indirect costs related to failures by resistant bacteria may have considerable influence [141]. In this sense, in a retrospective analysis of CAP treatment, significantly higher percentages of treatment failures were obtained in metropolitan areas with ≥25% resistance than in those with lower resistance rates, with an increase in 33% of costs in the areas with resistance rates higher than 25% [141, 142]. In a cost-effective analysis carried out in three countries, first-line treatment effective against the major CAP pathogens (including strains resistant to other antimicrobials) resulted in better clinical outcomes and lower treatment costs [143].

In addition to monetary costs, selection of resistance has a social cost derived from the impact of resistance in future infections in the community. The challenge for institutions and individual clinicians is to consider the potential impact of each antibiotic prescription on resistance [136]. Low potential of resistance selection and ecological consideration for human microbiota are two desirable features in antibiotic treatment [144]. Non-clinical studies carried out with cefditoren, which constitute the largest documentation in the ecological field for all antimicrobials, suggest that cefditoren possesses advantages with respect to its ability for countering diffusion and selection of resistance [145] in *S. pneumoniae* [78, 81] and *H. influenzae* [76, 79–82].

## Conclusions

Theoretically, the 15 years passed since its licensure should not have harmed the intrinsic activity of cefditoren against the respiratory human-adapted pathogens but have probably improved it somewhat since, maintaining its high activity against *H. influenzae* (regardless the increase of BLNAR and BLPACR isolates), *M. catarrhalis* and *S. pyogenes*, the percentage of penicillin-resistant *S. pneumoniae* has decreased, at least in countries where PCVs have been included in vaccination calendars. Due to this, *H. influenzae* has acquired more relevance, and the good activity of cefditoren against this species and its ability to counter diffusion of the *fts*I gene and the indirect pathogenicity by β-lactamase producing strains, strengths its value within the antibiotic armamentarium. Despite there is a lack of CLSI/EUCAST susceptibility breakpoints, the first decade of the present century has provided a plethora of pharmacodynamic studies with cefditoren, focused not only on activity/efficacy prediction but also on countering selection and diffusion of resistance, which support its role in the treatment of infections caused by respiratory human-adapted pathogens, thus filling the traditional gap remaining in antibiotic development [146] by an active post-marketing investigational task [147, 148].

## References

[1] Weimer KE, Juneau RA, Murrah KA, Pang B, Armbruster CE, Richardson SH (2011). Divergent mechanisms for passive pneumococcal resistance to β-lactam antibiotics in the presence of *Haemophilus influenzae*. J Infect Dis.

[2] Sorg RA, Lin L, van Doorn GS, Sorg M, Olson J, Nizet V (2016). Collective resistance in microbial communities by intracellular antibiotic deactivation. PLoS Biol.

[3] Lehtinen S, Blanquart F, Croucher NJ, Turner P, Lipsitch M, Fraser C (2017). Evolution of antibiotic resistance is linked to any genetic mechanism affecting bacterial duration of carriage. Proc Natl Acad Sci U S A.

[4] van Hecke Oliver, Wang Kay, Lee Joseph J., Roberts Nia W., Butler Chris C. (2017). Implications of Antibiotic Resistance for Patients’ Recovery From Common Infections in the Community: A Systematic Review and Meta-analysis. Clinical Infectious Diseases.

[5] García-Rey C, Fenoll A, Aguilar L, Casal J (2004). Effect of social and climatological factors on antimicrobial use and *Streptococcus pneumoniae* resistance in different provinces in Spain. J Antimicrob Chemother.

[6] Boccia D, Alegiani SS, Pantosti A, Moro ML, Traversa G (2004). The geographic relationship between the use of antimicrobial drugs and the pattern of resistance for *Streptococcus pneumoniae* in Italy. Eur J Clin Pharmacol.

[7] Cizman M, Pokorn M, Seme K, Orazem A, Paragi M (2001). The relationship between trends in macrolide use and resistance to macrolides of common respiratory pathogens. J Antimicrob Chemother.

[8] Gómez-Lus R, Granizo JJ, Aguilar L, Bouza E, Gutierrez A, García-de-Lomas J (1999). Is there an ecological relationship between rates of antibiotic resistance of species of the genus *Streptococcus*? The Spanish surveillance Group for Respiratory Pathogens. J Clin Microbiol.

[9] Granizo JJ, Aguilar L, Casal J, García-Rey C, Dal-Ré R, Baquero F (2000). *Streptococcus pneumoniae* resistance to erythromycin and penicillin in relation to macrolide and beta-lactam consumption in Spain (1979-1997). J Antimicrob Chemother.

[10] Granizo JJ, Aguilar L, Casal J, Dal-Ré R, Baquero F (2000). *Streptococcus pyogenes* resistance to erythromycin in relation to macrolide consumption in Spain (1986-1997). J Antimicrob Chemother.

[11] Pérez-Trallero E, García-de-la-Fuente C, García-Rey C, Baquero F, Aguilar L, dal-Ré R (2005). Geographical and ecological analysis of resistance, coresistance, and coupled resistance to antimicrobials in respiratory pathogenic bacteria in Spain. Antimicrob Agents Chemother.

[12] Baquero F, Baquero-Artigao G, Cantón R, García-Rey C (2002). Antibiotic consumption and resistance selection in *Streptococcus pneumoniae*. J Antimicrob Chemother.

[13] Gracia M, Díaz C, Coronel P, Gimeno M, García-Rodas R, Rodríguez-Cerrato V (2009). Antimicrobial susceptibility of *Streptococcus pyogenes* in central, eastern, and Baltic European countries, 2005 to 2006: the cefditoren surveillance program. Diagn Microbiol Infect Dis.

[14] Stefani S, Mezzatesta ML, Fadda G, Mattina R, Palù G, Rossano F (2008). Antibacterial activity of cefditoren against major community-acquired respiratory pathogens recently isolated in Italy. J Chemother.

[15] Tempera G, Furneri PM, Carlone NA, Cocuzza C, Rigoli R, Musumeci R (2010). Antibiotic susceptibility of respiratory pathogens recently isolated in Italy: focus on cefditoren. J Chemother.

[16] Pérez-Trallero E, Martín-Herrero JE, Mazón A, García-Delafuente C, Robles P, Iriarte V (2010). Antimicrobial resistance among respiratory pathogens in Spain: latest data and changes over 11 years (1996-1997 to 2006-2007). Antimicrob Agents Chemother.

[17] Smit PW, Lindholm L, Lyytikainen O, Jalava J, Patari-Sampo A, Vuopio J (2015). Epidemiology and emm types of invasive group a streptococcal infections in Finland, 2008-2013. Eur J Clin Microbiol Infect Dis.

[18] Villaseñor-Sierra A, Katahira E, Jaramillo-Valdivia AN, Barajas-García Mde L, Bryant A, Morfín-Otero R (2012). Phenotypes and genotypes of erythromycin-resistant *Streptococcus pyogenes* strains isolated from invasive and non-invasive infections from Mexico and the USA during 1999-2010. Int J Infect Dis.

[19] Chan JC, Chu YW, Chu MY, Cheung TK, Lo JY (2009). Epidemiological analysis of *Streptococcus pyogenes* infections in Hong Kong. Pathology.

[20] Lu B, Fang Y, Fan Y, Chen X, Wang J, Zeng J (2017). High prevalence of macrolide-resistance and molecular characterization of *Streptococcus pyogenes* isolates circulating in China from 2009 to 2016. Front Microbiol.

[21] Pfaller MA, Farrell DJ, Sader HS, Jones RN (2012). AWARE ceftaroline surveillance program (2008-2010): trends in resistance patterns among *Streptococcus pneumoniae*, *Haemophilus influenzae*, and *Moraxella catarrhalis* in the United States. Clin Infect Dis.

[22] Biedenbach DJ, Jones RN, Fritsche TR (2008). Antimicrobial activity of cefditoren tested against contemporary (2004-2006) isolates of *Haemophilus influenzae* and *Moraxella catarrhalis* responsible for community-acquired respiratory tract infections in the United States. Diagn Microbiol Infect Dis.

[23] Soriano F, Granizo JJ, Coronel P, Gimeno M, Ródenas E, Gracia M (2004). Antimicrobial susceptibility of *Haemophilus influenzae*, *Haemophilus parainfluenzae* and *Moraxella catarrhalis* isolated from adult patients with respiratory tract infections in four southern European countries. The ARISE project. Int J Antimicrob Agents.

[24] Càmara J, Marimón JM, Cercenado E, Larrosa N, Quesada MD, Fontanals D (2017). Decrease of invasive pneumococcal disease (IPD) in adults after introduction of pneumococcal 13-valent conjugate vaccine in Spain. PLoS One.

[25] Hays C, Vermee Q, Agathine A, Dupuis A, Varon E, Poyart C (2017). Demonstration of the herd effect in adults after the implementation of pneumococcal vaccination with PCV13 in children. Eur J Clin Microbiol Infect Dis.

[26] Regev-Yochay G, Katzir M, Strahilevitz J, Rahav G, Finn T, Miron D (2017). The herd effects of infant PCV7/PCV13 sequential implementation on adult invasive pneumococcal disease, six years post implementation; a nationwide study in Israel. Vaccine.

[27] Carnalla-Barajas MN, Soto-Noguerón A, Sánchez-Alemán MA, Solórzano-Santos F, Velazquez-Meza ME, Echániz-Aviles G (2017). Changing trends in serotypes of *S. pneumoniae* isolates causing invasive and non-invasive diseases in unvaccinated population in Mexico (2000-2014). Int J Infect Dis.

[28] D'Ancona F, Caporali MG, Del Manso M, Giambi C, Camilli R, D'Ambrosio F (2015). Invasive pneumococcal disease in children and adults in seven Italian regions after the introduction of the conjugate vaccine, 2008-2014. Epidemiol Prev.

[29] Ben-Shimol Shalom, Givon-Lavi Noga, Grisaru-Soen Galia, Megged Orli, Greenberg David, Dagan Ron (2018). Comparative incidence dynamics and serotypes of meningitis, bacteremic pneumonia and other-IPD in young children in the PCV era: Insights from Israeli surveillance studies. Vaccine.

[30] Gaviria-Agudelo CL, Jordan-Villegas A, Garcia C, Mc Cracken GH Jr. The Effect of 13-valent pneumococcal conjugate vaccine on the serotype distribution and antibiotic resistance profiles in children with invasive pneumococcal disease. J Pediatric Infect Dis Soc. 2016; Epub ahead of print. 10.1093/jpids/piw005 PMC7107452 26907814

[31] Picazo J, Ruiz-Contreras J, Casado-Flores J, Negreira S, García-de-Miguel MJ, Hernández-Sampelayo T (2013). Expansion of serotype coverage in the universal pediatric vaccination calendar: short-term effects on age- and serotype-dependent incidence of invasive pneumococcal clinical presentations in Madrid, Spain. Clin Vaccine Immunol.

[32] Fenoll A, Granizo JJ, Giménez MJ, Yuste J, Aguilar L (2015). Secular trends (1990-2013) in serotypes and associated non-susceptibility of *S. pneumoniae* isolates causing invasive disease in the pre−/post-era of pneumococcal conjugate vaccines in Spanish regions without universal paediatric pneumococcal vaccination. Vaccine.

[33] Tarragó D, Aguilar L, García R, Gimenez MJ, Granizo JJ, Fenoll A (2011). Evolution of clonal and susceptibility profiles of serotype 19A *Streptococcus pneumoniae* among invasive isolates from children in Spain, 1990 to 2008. Antimicrob Agents Chemother.

[34] Beall BW, Gertz RE, Hulkower RL, Whitney CG, Moore MR, Brueggemann AB (2011). Shifting genetic structure of invasive serotype 19A pneumococci in the United States. J Infect Dis.

[35] Reinert R, Jacobs MR, Kaplan SL (2010). Pneumococcal disease caused by serotype 19A: review of the literature and implications for future vaccine development. Vaccine.

[36] Kim L, McGee L, Tomczyk S, Beall B (2016). Biological and epidemiological features of antibiotic-resistant *Streptococcus pneumoniae* in pre- and post-conjugate vaccine eras: a United States perspective. Clin Microbiol Rev.

[37] Tomczyk S, Lynfield R, Schaffner W, Reingold A, Miller L, Petit S (2016). Prevention of antibiotic-nonsusceptible invasive pneumococcal disease with the 13-valent pneumococcal conjugate vaccine. Clin Infect Dis.

[38] Kaur R, Casey JR, Pichichero ME (2016). Emerging *Streptococcus pneumoniae* strains colonizing the nasopharynx in children after 13-valent pneumococcal conjugate vaccination in comparison to the 7-valent era, 2006-2015. Pediatr Infect Dis J.

[39] Kim SH, Song JH, Chung DR, Thamlikitkul V, Yang Y, Wang H (2012). Changing trends in antimicrobial resistance and serotypes of *Streptococcus pneumoniae* isolates in Asian countries: an Asian network for surveillance of resistant pathogens (ANSORP) study. Antimicrob Agents Chemother.

[40] Phongsamart W, Srifeungfung S, Chatsuwan T, Nunthapisud P, Treerauthaweeraphong V, Rungnobhakhun P (2014). Changing trends in serotype distribution and antimicrobial susceptibility of *Streptococcus pneumoniae* causing invasive diseases in Central Thailand, 2009-2012. Hum Vaccin Immunother.

[41] Biedenbach DJ, Jones RN (2009). Update of cefditoren activity tested against community-acquired pathogens associated with infections of the respiratory tract and skin and skin structures, including recent pharmacodynamic considerations. Diagn Microbiol Infect Dis.

[42] Tempera G, Furneri PM, Ferranti C, Genovese C, Ripa S, Ungheri S (2010). In vitro activity of cefditoren versus other antibiotics against *S. pneumoniae* clinical strains isolated in Italy. Int J Immunopathol Pharmacol.

[43] Fenoll A, Giménez MJ, Robledo O, Coronel P, Gimeno M, Casal J (2007). Activity of cefditoren against clinical isolates of *Streptococcus pneumoniae* showing non-susceptibility to penicillins, cephalosporins, macrolides, ketolides or quinolones. Int J Antimicrob Agents.

[44] Fenoll A, Aguilar L, Robledo O, Giménez MJ, Tarragó D, Granizo JJ (2007). Influence of the beta-lactam resistance phenotype on the cefuroxime versus cefditoren susceptibility of *Streptococcus pneumoniae* and *Haemophilus influenzae* recovered from children with acute otitis media. J Antimicrob Chemother.

[45] Fenoll A, Giménez MJ, Robledo O, Aguilar L, Tarragó D, Granizo JJ (2008). In vitro activity of oral cephalosporins against pediatric isolates of *Streptococcus pneumoniae* non-susceptible to penicillin, amoxicillin or erythromycin. J Chemother.

[46] Fritsche TR, Biedenbach DJ, Jones RN (2008). Update of the activity of cefditoren and comparator oral beta-lactam agents tested against community-acquired Streptococcus pneumoniae isolates (USA, 2004-2006). J Chemother.

[47] Seral C, Suárez L, Rubio-Calvo C, Gómez-Lus R, Gimeno M, Coronel P (2008). In vitro activity of cefditoren and other antimicrobial agents against 288 *Streptococcus pneumoniae* and 220 *Haemophilus influenzae* clinical strains isolated in Zaragoza, Spain. Diagn Microbiol Infect Dis.

[48] Fenoll A, Giménez MJ, Robledo O, Aguilar L, Tarragó D, Granizo JJ (2008). Influence of penicillin/amoxicillin non-susceptibility on the activity of third-generation cephalosporins against *Streptococcus pneumoniae*. Eur J Clin Microbiol Infect Dis.

[49] Lee MY, Ko KS, Oh WS, Park S, Lee JY, Baek JY (2006). In vitro activity of cefditoren: antimicrobial efficacy against major respiratory pathogens from Asian countries. Int J Antimicrob Agents.

[50] Yanagihara K, Watanabe A, Aoki N, Matsumoto T, Yoshida M, Sato J (2017). Nationwide surveillance of bacterial respiratory pathogens conducted by the surveillance committee of Japanese Society of Chemotherapy, the Japanese Association for Infectious Diseases, and the Japanese Society for Clinical Microbiology in 2012: general view of the pathogens’ antibacterial susceptibility. J Infect Chemother.

[51] Cafini F, Aguilar L, Alou L, Giménez MJ, Sevillano D, Torrico M (2008). Cidal activity of oral third-generation cephalosporins against *Streptococcus pneumoniae* in relation to cefotaxime intrinsic activity. Eur J Clin Microbiol Infect Dis.

[52] Mezzatesta ML, Gona F, Marchese G, Nicolosi D, Toscano MA, Stefani S (2009). Cefditoren versus community-acquired respiratory pathogens: time-kill studies. J Chemother.

[53] García-Cobos S, Campos J, Lázaro E, Román F, Cercenado E, García-Rey C (2007). Ampicillin-resistant non-beta-lactamase-producing *Haemophilus influenzae* in Spain: recent emergence of clonal isolates with increased resistance to cefotaxime and cefixime. Antimicrob Agents Chemother.

[54] Hotomi M, Fujihara K, Billal DS, Suzuki K, Nishimura T, Baba S (2007). Genetic characteristics and clonal dissemination of beta-lactamase-negative ampicillin-resistant *Haemophilus influenzae* strains isolated from the upper respiratory tract of patients in Japan. Antimicrob Agents Chemother.

[55] Barbosa AR, Giufrè M, Cerquetti M, Bajanca-Lavado MP (2011). Polymorphism in *fts*I gene and β-lactam susceptibility in Portuguese *Haemophilus influenzae* strains: clonal dissemination of beta-lactamase-positive isolates with decreased susceptibility to amoxicillin/clavulanic acid. J Antimicrob Chemother.

[56] Kim IS, Ki CS, Kim S, Oh WS, Peck KR, Song JH (2007). Diversity of ampicillin resistance genes and antimicrobial susceptibility patterns in *Haemophilus influenzae* strains isolated in Korea. Antimicrob Agents Chemother.

[57] Hasegawa K, Yamamoto K, Chiba N, Kobayashi R, Nagai K, Jacobs MR (2003). Diversity of ampicillin-resistance genes in *Haemophilus influenzae* in Japan and the United States. Microb Drug Resist.

[58] Setchanova LP, Kostyanev T, Alexandrova AB, Mitov IG, Nashev D, Kantardjiev T (2013). Microbiological characterization of *Streptococcus pneumoniae* and non-typeable *Haemophilus influenzae* isolates as primary causes of acute otitis media in Bulgarian children before the introduction of conjugate vaccines. Ann Clin Microbiol Antimicrob.

[59] Shiro H, Sato Y, Toyonaga Y, Hanaki H, Sunakawa K (2015). Nationwide survey of the development of drug resistance in the pediatric field in 2000-2001, 2004, 2007, 2010, and 2012: evaluation of the changes in drug sensitivity of *Haemophilus influenzae* and patients’ background factors. J Infect Chemother.

[60] García-Cobos S, Campos J, Cercenado E, Román F, Lázaro E, Pérez-Vázquez M (2008). Antibiotic resistance in *Haemophilus influenzae* decreased, except for beta-lactamase-negative amoxicillin-resistant isolates, in parallel with community antibiotic consumption in Spain from 1997 to 2007. Antimicrob Agents Chemother.

[61] García-Cobos S, Arroyo M, Pérez-Vázquez M, Aracil B, Lara N, Oteo J (2014). Isolates of β-lactamase-negative ampicillin-resistant *Haemophilus influenzae* causing invasive infections in Spain remain susceptible to cefotaxime and imipenem. J Antimicrob Chemother.

[62] Resman F, Ristovski M, Forsgren A, Kaijser B, Kronvall G, Medstrand P (2012). Increase of β-lactam-resistant invasive *Haemophilus influenzae* in Sweden, 1997 to 2010. Antimicrob Agents Chemother.

[63] Hu J, Wang XL, Xu F, Xie J, Liu HW, Yang LL (2015). Epidemiological survey of *Haemophilus influenzae*-positive hospitalized children: a retrospective analysis [Chinese]. Zhongguo Dang Dai Er Ke Za Zhi.

[64] García-Cobos S, Campos J, Román F, Carrera C, Pérez-Vázquez M, Aracil B (2008). Low beta-lactamase-negative ampicillin-resistant *Haemophilus influenzae* strains are best detected by testing amoxicillin susceptibility by the broth microdilution method. Antimicrob Agents Chemother.

[65] Sevillano D, Giménez MJ, Cercenado E, Cafini F, Gené A, Alou L (2009). Genotypic versus phenotypic characterization, with respect to beta-lactam susceptibility, of *Haemophilus influenzae* isolates exhibiting decreased susceptibility to beta-lactam resistance markers. Antimicrob Agents Chemother.

[66] Yang Q, Xu Y, Chen M, Wang H, Sun H, Hu Y (2012). In vitro activity of cefditoren and other comparators against *Streptococcus pneumoniae*, *Haemophilus influenzae*, and *Moraxella catarrhalis* causing community-acquired respiratory tract infections in China. Diagn Microbiol Infect Dis.

[67] Ulloa C, Guevara S, Soley C, Abdelnour A, Arguedas A (2014). In vitro activity of cefditoren against middle ear fluid isolates from Costa Rican children with otitis media. Chemotherapy.

[68] Tristram S, Jacobs MR, Appelbaum PC (2007). Antimicrobial resistance in *Haemophilus influenzae*. Clin Microbiol Rev.

[69] Soriano F, Giménez MJ, Aguilar L (2011). Cefditoren in upper and lower community-acquired respiratory tract infections. Drug Des Devel Ther.

[70] Barberán J, Aguilar L, Giménez MJ (2012). Update on the clinical utility and optimal use of cefditoren. Int J Gen Med.

[71] Aguilar L, Giménez MJ, Barberán J (2010). Drug resistance in community-acquired respiratory tract infections: role for an emerging antibacterial. Infect Drug Resist.

[72] Turnidge J, Paterson DL (2007). Setting and revising antibacterial susceptibility breakpoints. Clin Microbiol Rev.

[73] Cafini F, González N, Torrico M, Echeverría O, Sevillano D, Alou L (2006). Influence of the displacement of protein binding by ibuprofen in the activity of a third-generation cephalosporin against *Streptococcus pneumoniae* [article in Spanish]. Rev Esp Quimioter.

[74] Sevillano D, Giménez MJ, Alou L, Aguilar L, Cafini F, Torrico M (2007). Effects of human albumin and serum on the in vitro bactericidal activity of cefditoren against penicillin-resistant *Streptococcus pneumoniae*. J Antimicrob Chemother.

[75] Sevillano D, Aguilar L, Alou L, Giménez MJ, González N, Torrico M (2008). High protein binding and cidal activity against penicillin-resistant *S. pneumoniae*: a cefditoren in vitro pharmacodynamic simulation. PLoS One.

[76] Torrico M, Aguilar L, González N, Giménez MJ, Echeverría O, Cafini F (2007). Influence of TEM-1 beta-lactamase on the pharmacodynamic activity of simulated total versus free-drug serum concentrations of cefditoren (400 milligrams) versus amoxicillin-clavulanic acid (2,000/125 milligrams) against *Haemophilus influenzae* strains exhibiting an N526K mutation in the *fts*I gene. Antimicrob Agents Chemother.

[77] Sevillano D, Aguilar L, Alou L, Giménez MJ, González N, Echeverría O (2008). Beta-lactam activity against penicillin-resistant *Streptococcus pneumoniae* strains exhibiting higher amoxicillin versus penicillin minimum inhibitory concentration values: an in vitro pharmacodynamic simulation. Chemotherapy.

[78] Cafini F, Aguilar L, Sevillano D, Giménez MJ, Alou L, Fenoll A (2008). Decrease in bacterial load versus resistance selection of pneumococcal subpopulations by beta-lactam physiological concentrations over time: an in vitro pharmacodynamic simulation. Microb Drug Resist.

[79] Alou L, Giménez MJ, Sevillano D, Aguilar L, González N, Echeverría O (2007). Are beta-lactam breakpoints adequate to define non-susceptibility for all *Haemophilus influenzae* resistance phenotypes from a pharmacodynamic point of view?. J Antimicrob Chemother.

[80] González N, Aguilar L, Alou L, Giménez MJ, Sevillano D, Torrico M (2009). Influence of different resistance traits on the competitive growth of *Haemophilus influenzae* in antibiotic-free medium and selection of resistant populations by different β-lactams: an in vitro pharmacodynamic approach. J Antimicrob Chemother.

[81] Sevillano D, Aguilar L, Alou L, Giménez MJ, González N, Torrico M (2008). Beta-lactam effects on mixed cultures of common respiratory isolates as an approach to treatment effects on nasopharyngeal bacterial population dynamics. PLoS One.

[82] González N, Aguilar L, Sevillano D, Giménez MJ, Alou L, Cafini F (2011). Efficacy of simulated cefditoren versus amoxicillin-clavulanate free concentrations in countering intrastrain *fts*I gene diffusion in *Haemophilus influenzae*. Antimicrob Agents Chemother.

[83] Ramos-Sevillano E, Rodríguez-Sosa C, Cafini F, Giménez MJ, Navarro A, Sevillano D (2012). Cefditoren and ceftriaxone enhance complement-mediated immunity in the presence of specific antibodies against antibiotic-resistant pneumococcal strains. PLoS One.

[84] Cafini F, Yuste J, Giménez MJ, Sevillano D, Aguilar L, Alou L (2010). Enhanced in vivo activity of cefditoren in pre-immunized mice against penicillin-resistant *S. pneumoniae* (serotypes 6B, 19F and 23F) in a sepsis model. PLoS One.

[85] Granizo JJ, Sádaba B, Honorato J, Gimenez MJ, Sevillano D, Aguilar L (2008). Monte Carlo simulation describing the pharmacodynamic profile of cefditoren in plasma from healthy volunteers. Int J Antimicrob Agents.

[86] Wellington K, Curran MP (2004). Cefditoren pivoxil: a review of its use in the treatment of bacterial infections. Drugs.

[87] Food and Drug Administration. Spectracef prescribing information. https://www.accessdata.fda.gov/drugsatfda_docs/label/2005/021222s009lbl.pdf

[88] Lodise TP, Kinzig-Schippers M, Drusano GL, Loos U, Vogel F, Bulitta J (2008). Use of population pharmacokinetic modeling and Monte Carlo simulation to describe the pharmacodynamic profile of cefditoren in plasma and epithelial lining fluid. Antimicrob Agents Chemother.

[89] Craig WA (1998). Pharmacokinetic/pharmacodynamic parameters: rationale for antibacterial dosing of mice and men. Clin Infect Dis.

[90] Craig WA, Nightingale CH, Murakawa T, Ambrose PG (2001). Pharmacodynamics of antimicrobials: General concepts and applications. Antimicrobial pharmacodynamics in theory and clinical practice.

[91] Clinical and Laboratory Standards Institute (2009). Performance standards for antimicrobial susceptibility testing; nineteenth informational supplement. CLSI document M100-S19.

[92] Food and Drug Administration. Microbiology Data for Systemic Antibacterial Drugs — Development, Analysis, and presentation: Guidance for industry; 2016. https://www.fda.gov/downloads/Drugs/GuidanceComplianceRegulatoryInformation/Guidances/UCM182288.pdf

[93] Karlowsky JA, Jones ME, Draghi DC, Critchley IA, Thornsberry C, Sahm DF (2002). In vitro susceptibility of recent clinical isolates of pneumococci to the investigational cephalosporin cefditoren. Diagn Microbiol Infect Dis.

[94] Johnson DM, Biedenbach DJ, Beach ML, Pfaller MA, Jones RN (2000). Antimicrobial activity and in vitro susceptibility test development for cefditoren against *Haemophilus influenzae*, *Moraxella catarrhalis*, and *Streptococcus* species. Diagn Microbiol Infect Dis.

[95] Jones RN, Biedenbach DJ, Croco MA, Barrett MS (1998). In vitro evaluation of a novel orally administered cephalosporin (Cefditoren) tested against 1249 recent clinical isolates of *Haemophilus influenzae*, *Moraxella catarrhalis*, and *Streptococcus pneumoniae*. Diagn Microbiol Infect Dis.

[96] Agencia Española de Medicamentos y Productos Sanitarios. Meiact ficha técnica https://www.aemps.gob.es/cima/pdfs/es/ft/65975/FichaTecnica_65975.html.pdf

[97] Sádaba B, Azanza JR, Quetglas EG, Campanero MA, Honorato J, Coronel P (2007). Pharmacokinetic/pharmacodynamic serum and urine profile of cefditoren following single-dose and multiple twice- and thrice-daily regimens in healthy volunteers: a phase I study. Rev Esp Quimioter.

[98] Ball P, Baquero F, Cars O, File T, Garau J, Klugman K (2002). Antibiotic therapy of community respiratory tract infections: strategies for optimal outcomes and minimized resistance emergence. J Antimicrob Chemother.

[99] Granizo JJ, Giménez MJ, Barberán J, Coronel P, Gimeno M, Aguilar L (2006). The efficacy of cefditoren pivoxil in the treatment of lower respiratory tract infections, with a focus on the per-pathogen bacteriologic response in infections caused by *Streptococcus pneumoniae* and *Haemophilus influenzae*: a pooled analysis of seven clinical trials. Clin Ther.

[100] Granizo JJ, Giménez MJ, Barberán J, Coronel P, Gimeno M, Aguilar L (2008). Efficacy of cefditoren in the treatment of upper respiratory tract infections: a pooled analysis of six clinical trials. Rev Esp Quimioter.

[101] Kaplan EL, Tucker RM, Poling TL, Marsh D, Chou C (2001). A multicenter comparison of cefditoren pivoxil and penicillin VK. J Respir Dis.

[102] Gooch W, Marsh D, Slickler T, Hunt B (2000). Cefditoren is safe and effective treatment for streptococcal pharyngitis.

[103] Chow J, Russell M, Volk S, Chow C (2000). Efficacy of cefditoren pivoxil (CDTR) versus amoxicillin/clavulanate (AMX/CLV) in acute maxillary sinusitis (AMS).

[104] Alvarez-Sala JL, Kardos P, Martínez-Beltrán J, Coronel P, Aguilar L (2006). Clinical and bacteriological efficacy in treatment of acute exacerbations of chronic bronchitis with cefditoren-pivoxil versus cefuroxime-axetil. Antimicrob Agents Chemother.

[105] Ramírez JA, Tucker RM, Bettis RB, Cyganousky M, Hunt BJ (2001). Treating acute exacerbations of chronic bronchitis. J Resp Dis.

[106] Henry DC, Poling TL, Bettis RB, Junt BJ, Cyganouski M, Hom RC (2001). A double-blind, randomized study of cefditoren vs. cefuroxime for AECB. J Resp Dis.

[107] Blasi F, Tarsia P, Mantero M, Morlacchi LC, Piffer F (2013). Cefditoren versus levofloxacin in patients with exacerbations of chronic bronchitis: serum inflammatory biomarkers, clinical efficacy, and microbiological eradication. Ther Clin Risk Manag.

[108] Fogarty CM, Cyganowski M, Palo WA, Hom RC, Craig WA (2002). A comparison of cefditoren pivoxil and amoxicillin/ clavulanate in the treatment of community-acquired pneumonia: a multicenter, prospective, randomized, investigator-blinded, parallel-group study. Clin Ther.

[109] van Zyl L, le Roux JG, LaFata JA, Volk RS, Palo WA, Flamm R (2002). Cefditoren pivoxil versus cefpodoxime proxetil for community-acquired pneumonia: results of a multicenter, prospective, randomized, double-blind study. Clin Ther.

[110] Brook I (2017). Treatment challenges of group a Beta-hemolytic streptococcal pharyngo-tonsillitis. Int Arch Otorhinolaryngol.

[111] Camilli R, Vescio MF, Giufrè M, Daprai L, Garlaschi ML, Cerquetti M (2015). Carriage of *Haemophilus influenzae* is associated with pneumococcal vaccination in Italian children. Vaccine.

[112] Medaney F, Dimitriu T, Ellis RJ, Raymond B (2016). Live to cheat another day: bacterial dormancy facilitates the social exploitation of β-lactamases. ISME J.

[113] Budhani RK, Struthers JK (1998). Interaction of *Streptococcus pneumoniae* and *Moraxella catarrhalis*: investigation of the indirect pathogenic role of beta-lactamase-producing moraxellae by use of a continuous-culture biofilm system. Antimicrob Agents Chemother.

[114] Lewnard JA, Huppert A, Givon-Lavi N, Pettigrew MM, Regev-Yochay G, Dagan R (2016). Density, serotype diversity, and fitness of *Streptococcus pneumoniae* in upper respiratory tract cocolonization with nontypeable *Haemophilus influenzae*. J Infect Dis.

[115] Weimer KE, Armbruster CE, Juneau RA, Hong W, Pang B, Swords WE (2010). Coinfection with *Haemophilus influenzae* promotes pneumococcal biofilm formation during experimental otitis media and impedes the progression of pneumococcal disease. J Infect Dis.

[116] Prince AS (2002). Biofilms, antimicrobial resistance, and airway infection. N Engl J Med.

[117] Dy R, Sethi S (2016). The lung microbiome and exacerbations of COPD. Curr Opin Pulm Med.

[118] Shukla SD, Fairbairn RL, Gell DA, Latham RD, Sohal SS, Walters EH (2016). An antagonist of the platelet-activating factor receptor inhibits adherence of both nontypeable *Haemophilus influenzae* and *Streptococcus pneumoniae* to cultured human bronchial epithelial cells exposed to cigarette smoke. Int J Chron Obstruct Pulmon Dis.

[119] Bellinghausen C, Gulraiz F, Heinzmann AC, Dentener MA, Savelkoul PH, Wouters EF (2016). Exposure to common respiratory bacteria alters the airway epithelial response to subsequent viral infection. Respir Res.

[120] Swords WE (2012). Nontypeable *Haemophilus influenzae* biofilms: role in chronic airway infections. Front Cell Infect Microbiol.

[121] Murphy TF, Kirkham C, Sethi S, Lesse AJ (2005). Expression of a peroxiredoxin-glutaredoxin by *Haemophilus influenzae* in biofilms and during human respiratory tract infection. FEMS Immunol Med Microbiol.

[122] Ahearn CP, Gallo MC, Murphy TF. Insights on persistent airway infection by non-typeable *Haemophilus influenzae* in chronic obstructive pulmonary disease. Pathog Dis. 2017;75(4). 10.1093/femspd/ftx042 PMC5437125 28449098

[123] Pang B, Hong W, West-Barnette SL, Kock ND, Swords WE (2008). Diminished ICAM-1 expression and impaired pulmonary clearance of nontypeable *Haemophilus influenzae* in a mouse model of chronic obstructive pulmonary disease/emphysema. Infect Immun.

[124] Sethi S (2010). Infection as a comorbidity of COPD. Eur Respir J.

[125] Roveta S, Schito AM, Marchese A, Schito GC (2007). Activity of moxifloxacin on biofilms produced in vitro by bacterial pathogens involved in acute exacerbations of chronic bronchitis. Int J Antimicrob Agents.

[126] Maestre JR, Mateo M, Méndez ML, Aguilar L, Gimenez MJ, Alou L (2010). In vitro interference of beta-lactams with biofilm development by prevalent community respiratory tract isolates. Int J Antimicrob Agents.

[127] Di Marco F, Braido F, Santus P, Scichilone N, Blasi F. The role of cefditoren in the treatment of lower community-acquired respiratory tract infections (LRTIs): from bacterial eradication to reduced lung inflammation and epithelial damage. Eur Rev Med Pharmacol Sci 2014;18:321–332. 24563430

[128] Duell BL, Su YC, Riesbeck K (2016). Host-pathogen interactions of nontypeable *Haemophilus influenzae*: from commensal to pathogen. FEBS Lett.

[129] Sethi S (2004). Bacteria in exacerbations of chronic obstructive pulmonary disease: phenomenon or epiphenomenon?. Proc Am Thorac Soc.

[130] Pragman AA, Berger JP, Williams BJ (2016). Understanding persistent bacterial lung infections: clinical implications informed by the biology of the microbiota and biofilms. Clin Pulm Med.

[131] Domenech A, Ardanuy C, Pallares R, Grau I, Santos S, De la Campa AG (2013). Some pneumococcal serotypes are more frequently associated with relapses of acute exacerbations in COPD patients. PLoS One.

[132] Canut A, Martín-Herrero JE, Labora A, Maortua H (2007). What are the most appropriate antibiotics for the treatment of acute exacerbation of chronic obstructive pulmonary disease? A therapeutic outcomes model. J Antimicrob Chemother.

[133] Pettigrew MM, Tsuji BT, Gent JF, Kong Y, Holden PN, Sethi S (2016). Effect of fluoroquinolones and macrolides on eradication and resistance of *Haemophilus influenzae* in chronic obstructive pulmonary disease. Antimicrob Agents Chemother.

[134] Blasi F, Concia E, Del Prato B, Giusti M, Mazzei T, Polistena B (2017). The most appropriate therapeutic strategy for acute lower respiratory tract infections: a Delphi-based approach. J Chemother.

[135] Mandell LA, Wunderink RG, Anzueto A, Bartlett JG, Campbell GD, Dean NC (2007). Infectious Diseases Society of America/American Thoracic Society consensus guidelines on the management of community-acquired pneumonia in adults. Clin Infect Dis.

[136] Bosso JA, Drew RH (2011). Application of antimicrobial stewardship to optimise management of community acquired pneumonia. Int J Clin Pract.

[137] González-Castillo J, Martín-Sánchez FJ, Llinares P, Menéndez R, Mujal A, Navas E (2014). Guidelines for the management of community-acquired pneumonia in the elderly patient. Rev Esp Quimioter.

[138] Menendez R, Montull B, Mendez R. Antibiotic choice, route and duration: minimizing the harm associated with antibiotics. In: Chalmers JD, Pletz MW, Aliberti S, editors. Community-Acquired Pneumonia. Eur Respir Mon. 2014. p. 155–67.

[139] Barberán J, Mensa J (2009). Cefditoren and community-acquired lower respiratory tract infections [article in Spanish]. Rev Esp Quimioter.

[140] Athanassa Z, Makris G, Dimopoulos G, Falagas ME (2008). Early switch to oral treatment in patients with moderate to severe community-acquired pneumonia: a meta-analysis. Drugs.

[141] Asche C, McAdam-Marx C, Seal B, Crookston B, Mullins CD (2008). Treatment costs associated with community-acquired pneumonia by community level of antimicrobial resistance. J Antimicrob Chemother.

[142] Tillotson GS, Zinner SH (2017). Burden of antimicrobial resistance in an era of decreasing susceptibility. Expert Rev Anti-Infect Ther.

[143] Martin M, Quilici S, File T, Garau J, Kureishi A, Kubin M (2007). Cost-effectiveness of empirical prescribing of antimicrobials in community-acquired pneumonia in three countries in the presence of resistance. J Antimicrob Chemother.

[144] Granizo JJ, Rodicio MP. Ecology, antibiotics and biodiversity. In: SiCom XXISL, editor. From microbiology to clinical practice: the cefditoren case. Madrid; 2009. p. 225–35.

[145] Giménez MJ, Aguilar L. Can ecology be a parameter for antibiotic election? In: SiCom XXISL, editor. From microbiology to clinical practice: the cefditoren case. Madrid; 2009. p. 253–9.

[146] Aguilar L, Giménez MJ (2008). Gaps in antibiotic development: the post-marketing task. Rev Med Microbiol.

[147] From microbiology to clinical practice: The cefditoren case. SiCom XXI SL: Madrid, 2009.

[148] From microbiology to clinical practice: The cefditoren case II. SiCom XXI SL, Madrid;2012.

